# ERK1 and ERK2 Map Kinases: Specific Roles or Functional Redundancy?

**DOI:** 10.3389/fcell.2016.00053

**Published:** 2016-06-08

**Authors:** Roser Buscà, Jacques Pouysségur, Philippe Lenormand

**Affiliations:** ^1^Centre National de la Recherche Scientifique UMR7284, Institut National de la Santé et de la Recherche Médicale, Centre A. Lacassagne, Institute for Research on Cancer and Ageing of Nice, University of Nice-Sophia AntipolisNice, France; ^2^Centre Scientifique de MonacoMonaco, Monaco

**Keywords:** intracellular signaling, MAP kinases, ERK1 and ERK2 isoforms, gene silencing, gene disruption, expression of isoforms in vertebrates, protein sequence evolution

## Abstract

The MAP kinase signaling cascade Ras/Raf/MEK/ERK has been involved in a large variety of cellular and physiological processes that are crucial for life. Many pathological situations have been associated to this pathway. More than one isoform has been described at each level of the cascade. In this review we devoted our attention to ERK1 and ERK2, which are the effector kinases of the pathway. Whether ERK1 and ERK2 specify functional differences or are in contrast functionally redundant, constitutes an ongoing debate despite the huge amount of studies performed to date. In this review we compiled data on ERK1 vs. ERK2 gene structures, protein sequences, expression levels, structural and molecular mechanisms of activation and substrate recognition. We have also attempted to perform a rigorous analysis of studies regarding the individual roles of ERK1 and ERK2 by the means of morpholinos, siRNA, and shRNA silencing as well as gene disruption or gene replacement in mice. Finally, we comment on a recent study of gene and protein evolution of ERK isoforms as a distinct approach to address the same question. Our review permits the evaluation of the relevance of published studies in the field especially when measurements of global ERK activation are taken into account. Our analysis favors the hypothesis of ERK1 and ERK2 exhibiting functional redundancy and points to the concept of the global ERK quantity, and not isoform specificity, as being the essential determinant to achieve ERK function.

## Introduction

The Ras/Raf/MEK/ERK cascade is a key signaling pathway which integrates extracellular clues from cell surface receptors to gene expression and regulation of multiple cellular proteins. ERK cascade plays a crucial role in multiple cellular processes such as cell proliferation, differentiation, adhesion, migration and survival. Therefore, it is essential for many physiological events including development, immunity, metabolism, and memory formation. The core of this pathway consists in activation of the cascade of three kinases Raf, MEK, and ERK. Raf and MEK are described to date as cytoplasmic kinases with a single well established substrate, however ERK is unique in this cascade as it phosphorylates multiple substrates in all cellular compartments (at least 270 substrates have been identified in proteomics screening von Kriegsheim et al., 2009). MEK and ERK can also be activated independently of Raf by the COT/TPL2 kinase (Johannessen et al., 2010) and by mos during meiotic maturation (Nebreda et al., 1993). Integration of ERK signaling can also proceed from the regulation of scaffolding proteins which function mainly to bring several members of the cascade into close vicinity in order to increase the efficiency and strength of activation (reviewed in Roskoski, 2012; Cseh et al., 2014). Multiple isoforms have been described at each step of the Ras/Raf/MEK/ERK pathway (reviewed in Lefloch et al., 2009). At the levels of Raf and MEK, functional differences and tissue-specific expression among isoforms have been clearly established (see Section Search for Specific Functions of ERK Isoforms). On the other hand, ERK1 and ERK2 seem to be expressed ubiquitously and there are no obvious regulatory differences inferred from their protein sequences, their regulation or their sub-cellular localization. The aim of this review is to put into perspective for the first time the vast body of work that has attempted to find differential roles for ERK1 and ERK2 or tried to demonstrate their functional redundancy.

Prior to scrutinizing studies on ERK isoforms functions, we will recap the main traits of ERK1/2 regulation, action and role to aid in understanding the studies on isoform functions.

### Overview on ERK signaling

Before the molecular cloning of ERKs by Melanie Cobb's group (Boulton et al., 1990), ERK1 and ERK2 were known as two proteins respectively p44 and p42 MAPK rapidly phosphorylated in response to all mitogens (Kohno and Pouysségur, 1986; Sturgill et al., 1988). The essentiality of ERK signaling for cell proliferation of mammalian fibroblasts was first demonstrated by expression of antisense cDNAs or dominant negative mutants which inhibited global ERK activity (Pagès et al., 1993). Later, disruption of the *erk2* isoform was shown to induce early embryonic death (Hatano et al., 2003; Saba-El-Leil et al., 2003; Yao et al., 2003). In adult mice invalidation of both isoforms, led to animal death within 3 weeks by multiple organ failures (Blasco et al., 2011). Collectively, these experiments demonstrate the absolute requirement for a minimal ERK expression to permit proliferation and mammalian life.

Using quantitative proteomics, 284 ERK-interacting proteins have been identified, and 60 of these proteins changed their own binding to ERK upon induction of differentiation (von Kriegsheim et al., 2009). ERKs phosphorylate serine or threonine residues of substrates on the sequence PXS/TP. Many proteins possess this sequence and are not *bona fide* ERK substrates. Specificity is provided by docking motifs located at the back of the kinase (graphical representation on 3D structure in Busca et al. (2015). These docking interactions have been proposed to increase the local concentration of substrates to favor their phosphorylation when ERK is active. Two motifs on ERKs bind to substrates, 16 amino-acids that constitute the common docking site (CDS) also called D-recruitment site (DRS), and 7 amino-acids that constitute the F-recruitment site (FRS). The DEJL motif of substrates (*d*ocking site for *E*RK and *J*NK, *L*XL also called KIM, kinase interacting motif) binds to the DRS (Lee et al., 2004), whereas the DEF (FXFP) motif of substrates binds to the FRS (Liu et al., 2006). The duration of ERK activation can lead to phosphorylation of waves of substrates according to their docking sites content (Murphy et al., 2004). Of great importance for cell fate, ERKs phosphorylate multiple transcription factors, hence active ERKs translocate to the nucleus (Lenormand et al., 1993) mainly by passive diffusion. This ERK nuclear translocation is certainly a key process in ERK signaling. Indeed we demonstrated that retention of active ERK in the cytoplasm abolishes cell cycle progression and the onset of DNA replication (Brunet et al., 1999). ERK nuclear translocation is favored by ERKs binding to the FXFG motif of nucleoporins (Whitehurst et al., 2002), a motif that mimics the FXFP motif of ERKs substrates. Ideed, recently it was shown that ERK-mediated phosphorylation of nucleoporins regulates ERK translocation (Shindo et al., 2016). Others have indicated that ERKs entry into the nucleus may require active transport dependent on Ran, especially when ERKs are fused to beta-galactosidase (Adachi et al., 2000). ERK nuclear accumulation of ERKs requires synthesis of nuclear anchors (Lenormand et al., 1998), however nuclear translocation of ERKs can be regulated by the abundance of interactor proteins, for example over-expression of PEA-15 can sequester ERK in the cytoplasm (Formstecher et al., 2001). Similarly, it was demonstrated that Sef binds to activated forms of MEK, inhibits the dissociation of the MEK–ERK complex, and blocks nuclear translocation of activated ERK (Torii et al., 2004). PEA-15 and Sef expression do not prevent phosphorylation of cytoplasmic substrates by ERK, however they block activation of nuclear substrates.

MEK activates ERKs by dual phosphorylation on the threonine and tyrosine residues of the sequence T^185^EY^187^ (sequence of human ERK2). Inactivation of ERKs requires the removal of either one or both sites of the TEY motif. Tyrosine-specific phosphatases include PTP-SL, STEP, and HePTP whereas the threonine-specific phosphatases include protein phosphatase 2A and 2C. A large family of dual specificity phosphatases, the DUSPs, can inactivate ERKs. A coordinated action of all these phosphatases induced by ERK is required to shape the temporal ERK activity for proper mammalian development. Invalidation of a single ERK phosphatase such as DUSP6 can be sufficient to increase the basal ERKs phosphorylation level (Li et al., 2007; Maillet et al., 2008). However, DUSP6 invalidation may lead to minor cardiac abnormalities (Maillet et al., 2008) or a range of phenotypes in the same litter such as embryonic death, cranio-facial abnormalities or lack of obvious phenotype (Li et al., 2007). Hence it is difficult to sort out the precise role of each phosphatase to regulate the activation level of ERKs during development and adult homeostasis. Interestingly, in some cases such as the invalidation of DUSP5, phenotypes appear only upon challenging the pathway. Lack of DUSP5 in mouse embryo fibroblasts leads to increase nuclear phospho ERKs content, and lack of DUSP5 in mouse increases sensitivity to mutant Harvey-Ras (HRasQ61L)-driven papilloma (Rushworth et al., 2014). As described above, ERKs nuclear accumulation requires protein neo-synthesis; in fact some of these nuclear anchors are phosphatases since ERK accumulates in the nucleus in the dephosphorylated form (Volmat et al., 2001). DUSP5 is one of the phosphatases that drive ERK nuclear accumulation and dephosphorylation (Mandl et al., 2005).

### Pathological consequences of abnormal ERK signaling

ERK pathway is misregulated *via* germline mutations in genes that encode components or regulators of the cascade, causing disease such as type1 neurofibromatosis and Noonan syndrome (pathologies clustered under the name rasopathies, Rauen, 2013). ERK pathway is also over-activated in many cancers. For example, at the receptor level, the HER2/Neu (EGF family) oncogene can be over-expressed or mutated leading to persistent activation of the pathway (Menard et al., 2004). Similarly EGFR receptor is often mutated in lung and colon cancers (Barber et al., 2004). At the level of Ras, point activating mutations of K-Ras are found in over 95% of pancreatic ductal adenocarcinomas for example (Bryant et al., 2014). Downstream of Ras, the B-Raf kinase is also mutated in many cancers such as at least 66% of melanomas (e.g., mutation B-Raf^V600E^; Davies et al., 2002). At the level of MEK, somatic mutations have been found via next-generation sequencing of tumoral tissues. Interestingly, in the langerhans cell histiocytosis disease usually B-Raf is mutated; however when B-Raf is not mutated, MEK1 is activated by mutations in 50% of remaining cases (Brown et al., 2014), highlighting again the importance of ERK pathway in oncogenesis. It has also been shown that up to 8% of melanomas present activating mutations of MEK1 or MEK2 (Nikolaev et al., 2012). At the ERK level, amplification of the ERK2 gene has been found in tumors of patients treated with anti EGF-receptor kinase inhibitors. This amplification has been proposed to contribute to the treatment resistance (Ercan et al., 2012). On the contrary, loss of small chromosomal segment encompassing one allele of ERK2 has been observed in children that exhibit microcephaly, impaired cognition, and developmental delay (Samuels et al., 2008).

The very high prevalence of human cancers harboring constitutive activation of the ERK pathway has prompted a massive development of pharmacological inhibitors targeting members of the ERK cascade. After the great success of the tyrosine kinase inhibitor Imatinib to treat chronic myeloid leukemia (CML) by blocking the kinase activity of BCR-ABL, the hope was to obtain similar results when treating many cancers where the driver mutation was in the ERK kinase-cascade. With kinase inhibitors, CML patients are expected to have a normal life-expectancy (Jabbour, 2016). However, for cancers arising from mutations in ERK pathway, patients with mutations at the levels of receptors (HER/Neu, PDGF, and EGF-receptors) relapse after some months of treatment. At the level of Ras, targeted therapies have tried to block the anchoring of Ras at the plasma membrane unsuccessfully (Baines et al., 2011). At the level of Raf, inhibitors of the activated form of B-Raf have been approved recently to treat melanoma, for example PLX4032 (Bollag et al., 2010). Initially, most patients display dramatic improvements of the tumor burden, however rapidly resistances to the treatment arise, leading in most cases to reactivation of ERK pathway. Resistance can occur from amplification of tyrosine kinase receptors, acquisition of mutations in N-Ras, amplification of mutant B-Raf, alternative splicing of mutant B-Raf or even mutations in MEK protein. Moreover, it has been shown that inhibitors of B-Raf^V600E^ mutant can paradoxically activate the ERK pathway, especially in the presence of oncogenic Ras. This was unexpected since B-Raf acts downstream of Ras. However, B-Raf inhibitors drive the formation of B-Raf/C-Raf hetero dimers, where the drug-bound partner drives activation of the drug-free partner through scaffolding or conformational modifications leading to paradoxical activation of cRAF (Poulikakos et al., 2010; Hatzivassiliou et al., 2012). Medical hope in this field lays in pan-Raf inhibitors that target also Src-family of kinases and block all types of Raf dimers (Girotti et al., 2015) or combined therapies, for example with inhibitors at other levels of the ERK cascade.

MEK is mainly activated by Raf, however in some cells MEK has also been described to be activated by the TPL2/Cot pathway. Therefore, tumors have been shown to escape Raf inhibitors by re-activating TPL2/Cot (Johannessen et al., 2010). Furthermore, it has been shown that ERK can retro-phosphorylate MEK1 on threonine 292 to reduce its activation (Mansour et al., 1994; Saito et al., 1994), therefore any decrease of Raf activity by inhibitors, diminishes ERK activity, and as a loop, this decreased ERK activity reduces retro-inhibition of MEK therefore stabilizing a threshold of ERK activation. Considering these retro-controls, it was thought that MEK inhibitors would be better candidates to target cancers driven by activating mutations in ERK pathway. Interestingly, the activating lip of MEK kinase is uniquely structured allowing the design of very potent and specific kinase inhibitors (Ohren et al., 2004). Two families of MEK inhibitors have been designed: allosteric inhibitors acting via binding to the activating lip (e.g., PD184352) and more recently, classical competitors of ATP-binding that block MEK activity (e.g., E6201, Narita et al., 2014). Unfortunately, these inhibitors generate secondary effects in patients and therefore a reduction of dosage or duration of treatment is imposed, hence after an initial effectiveness, tumors relapse. Secondary effects observed during treatment with MEK inhibitors encompass hand and foot rash, diarrhea, nausea, retinopathy, visual disturbance, mental status change, alopecia, stomatitis, and verrucous keratoses (Welsh and Corrie, 2015). In 2013 the MEK inhibitor Trametinib was approved for the treatment of melanoma patients with unresectable or metastatic melanoma with BRAF mutations. As of 2016, many clinical trials are ongoing with several MEK inhibitors demonstrating the endeavor to cure cancers and rasopathies (for example: selumetinib/AZD6244; MEK162; Refametinib/BAY86-9766; Trametinib/ GSK1120212; GDC-0973; PD-0325901; RO5126766 and Cobimetinib/GDC-0973).

The resistance to treatments from Raf and MEK inhibitors has led to clinical trials for combined therapy such treatment with dabrafenib and trametinib for un-resectable or metastatic melanoma with a B-Raf^V600E^ or B-Raf^V600K^ mutations. Nevertheless, the majority of patients face relapse even with the combination of treatments by mechanisms which are not yet understood (reviewed by Queirolo et al., 2015).

Resistance to treatment by inhibitors of RTKs, Raf and MEK have finally led to target ERK itself. Interestingly even cells resistant to MEK or Raf inhibition (for example by mutations in the kinase pocket of MEK or via amplification of K-Ras expression) were shown to be sensitive to ERK kinase inhibition (Hatzivassiliou et al., 2012; Morris et al., 2013). Specific ERK inhibitors are very difficult to design due to the high homology between ERK and CDK kinases pockets (these kinases belong to the same family of CMGC kinases Manning et al., 2002). For example, the CDK2 inhibitor purvalanol was shown to block activation of ERK at similar concentrations *in-cellulo* despite a higher affinity for purified CDK1/2 protein (Knockaert et al., 2002). As of 2016, five clinical assays are ongoing with four ERK inhibitors (MK-8353/SCH900353, BVD 523, RG7842/GDC0994, and CC-90003). No conclusive results have been reached up to date but these trials bring new and promising hope.

## Origin of ERK isoforms

**Figure 1:**
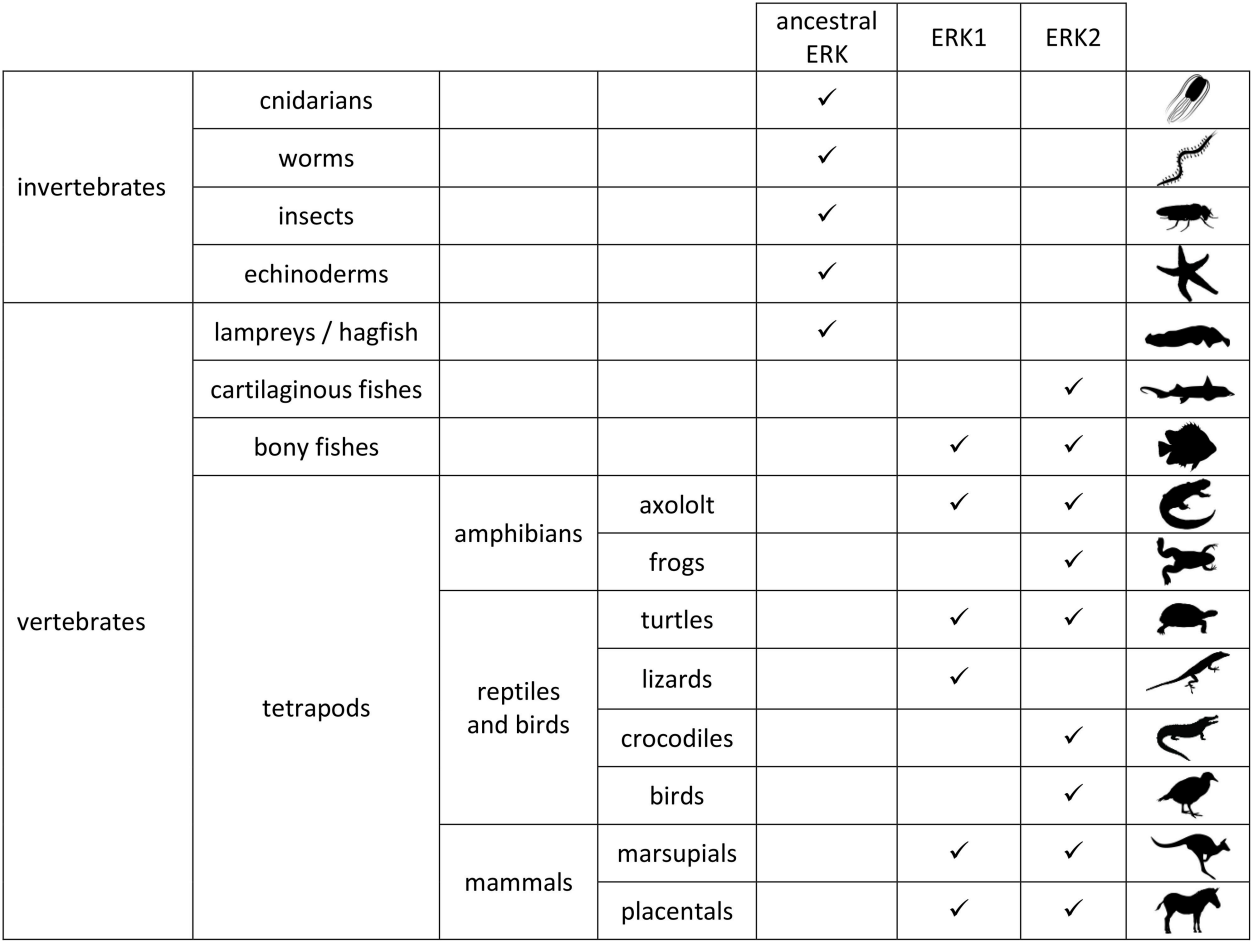
**Expression of ERK proteins in animals**. ERK1 and ERK2 proteins were classified as such upon phylogenic study of amino-acid coding sequences (same conclusions were reached with nucleotide sequences; Busca et al., 2015). In invertebrates only one *erk* gene was identified so far. Ancestral ERK corresponds to ERK protein sequence that cannot be classified into ERK1 or ERK2 group. Protein expression in vertebrate brains was described in the same study. Animal silhouettes are from phylopic (http://phylopic.org/).

In 1991 two isoforms of ERK were discovered in mammals: ERK1 (MAPK3) and ERK2 (MAPK1) (Boulton et al., 1991). At that time, two MAP kinases similar to mammalian ERK were discovered in budding yeast (S. cerevisiae), FUS3 and KSS1 (Courchesne et al., 1989; Elion et al., 1990) hence one could consider that two kinase isoforms were necessary from yeast to humans. However, only one kinase similar to ERK was discovered in fission yeast (S. pombe), spk1 (Toda et al., 1991). It is now established that mammalian ERK1/2 isoforms arose independently from budding yeast MAPK isoforms, therefore the comparative studies between FUS3 and KSS1 cannot be transposed to ERK1 and ERK2 studies. Indeed a yeast-specific whole genome duplication (WGD) led to emergence of FUS3 and KSS1 (Wolfe et al., 2015), whereas the vertebrate-specific WGD led to the emergence of ERK1 and ERK2. WGDs are essential events in the evolution that have been observed in all phylla of life, whereby an organism possesses initially two copies of its entire genome. After the duplication event, duplicate genes can have different fates that participate in speciation as described for paramecium (Aury et al., 2006). The origin of the duplication leading to emergence of ERK1 and ERK2 was determined to be early in the vertebrate phylum (Busca et al., 2015). First of all, in all tetrapod clades at least one animal expresses two ERKs that can be phylogenically classified into ERK1 and ERK2 by their protein and nucleic sequences. It is the case for all mammals analyzed so far, for turtles in the reptilian clade and for axolotl in amphibian clade. Among fishes, all teleost fishes express two ERKs and it has been shown that the most ancient branch of ray-finned fishes, the bichir, express ERK1 and ERK2. Demonstration of ERK1 and ERK2 presence at this evolutionary node is important since bichirs diverged from other vertebrates prior to teleost fishes that underwent an additional WGD. Hence ERK1 and ERK2 arose at least 400 million years ago and studies of ERK isoforms in fishes are relevant to mammalian ERK isoforms since they arose from the same duplication event (Busca et al., 2015). Further away in the vertebrate evolution tree, members of all classes of cartilaginous fishes express only one ERK which could be classified into the ERK2 group by three independent topological methods used to infer the phylogeny. At the base of vertebrates, hagfish and lampreys, belonging to the two most divergent vertebrate species compared to mammals, also express only one ERK isoform that is not possible to be classified as ERK1 or ERK2. In all invertebrates studied so far, only one ancestral ERK was identified; hence it is concluded that ERK1 and ERK2 arose from the early WGD at the base of the vertebrate phylogenic tree (representation in Figure 1). From ray-finned fishes to humans, ERK isoforms can be easily classified into ERK1 or ERK2 groups according to their coding sequences.

## Comparing structures and regulation of ERK isoforms

### Gene structure

In vertebrates, *erk2* gene is larger than *erk1* gene, this is especially obvious in mammals where *erk1* genes are on average 15 fold smaller than *erk2* genes in the same animal (sizes calculated from ATG initiating codon to stop codon; Busca et al., 2015). The first intron of mammalian *erk2* and the second intron of drosophila *erk* are very large (59 kb and 25 kb respectively). Clearly in drosphila, *erk* intron is among the largest ones, and requires specific factors for proper splicing (Ashton-Beaucage et al., 2010; Roignant and Treisman, 2010). These huge introns could provide a unique regulation during development. This point should be better understood in the future by genomic truncation of the mouse *erk2* gene's first intron for example.

### Alternative splicing in the coding sequence

R. Seger and his team reported that the intron 7 of several mammalian ERK1s is not always spliced properly. In rat genome the intron 7 sequence, if not spliced, is in phase with the coding sequence of exons 7 and 8, thereby increasing the size of ERK1 by 26 amino-acids to a predicted protein size of 45.8 kD. Indeed, a signal corresponding to a larger ERK protein has been detected in rat IEC-6 cells by western blot (Boucher et al., 2004). These ERK1 alternative splices were named ERK1b in rat and ERK1c in human and chimpanzee. Human ERK1c predicted size is 40.1 kD instead of 43.1 kD for the normally spliced ERK1 since non-splicing of intron 7 introducing a stop codon. Considering isoforms functionality, it has been reported that ERK1c mediates cell density-induced Golgi fragmentation (Aebersold et al., 2004).

**Figure 2:**
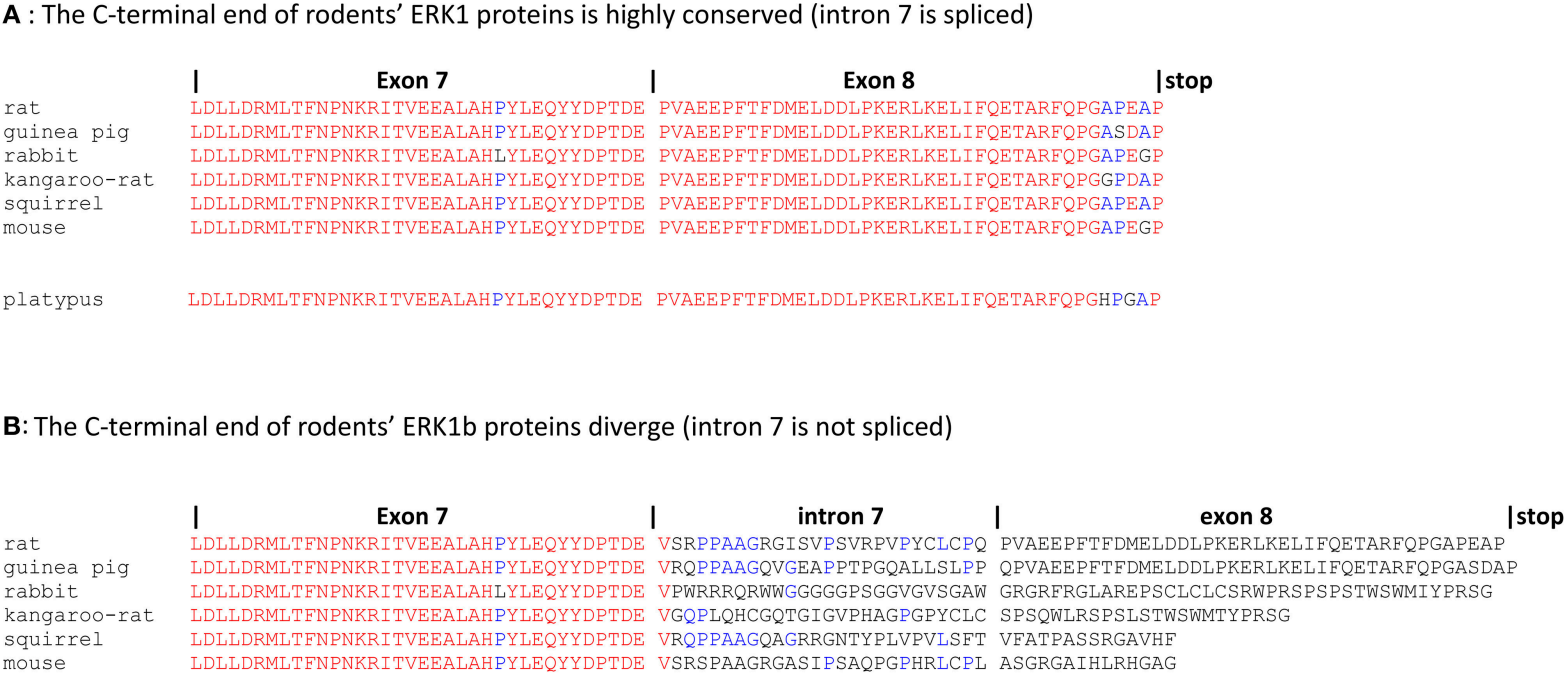
*****erk1b*** RNAs from related species drive synthesis of unrelated proteins domains**. Genomic sequences corresponding to exon7-intron7-exon8 of MAPK3 (*erk1*) were retrieved from Ensembl release 83. First line: extension of the introns and exons, separated by vertical bars. “stop” indicates the final of ERK1 and ERK1b coding sequences. Sequences were aligned by multalin program with identity matrix (Corpet, 1988). Alignments were performed also with Tcoffee and Clustal Omega with the same results (not shown). Rat (*Rattus norvegicus*), guinea pig (*Cavia porcellus*), rabbit (*Oryctolagus cuniculus*), kangaroo-rat (*Dipodomys ordii*), squirrel (*Ictidomys tridecemlineatus*), mouse (*Mus musculus*), platypus (*Ornithorhynchus anatinus*). Letters in red, amino-acids highly conserved for a position among the protein sequences; letters in blue, amino-acids showing limited conservation; letters in black, amino-acids showing no conservation in the aligned sequences. **(A)** The C-terminal sequences of rodents' ERK1 proteins are highly conserved; platypus'ERK1 protein sequence is highly similar to rodents' ERK1s. **(B)** Rodents' ERK1b proteins display no significant conserved protein motifs after exon7.

It is tempting to wonder whether these alternative spliced forms provide a ground to explain why ERK1 and ERK2 isoforms have different functions and have been kept after the WGD. We consider that this is very unlikely for several reasons. First, ERK1b and ERK1c splice variants are expressed at very low levels (Aebersold et al., 2004; Boucher et al., 2004); secondly it is extremely puzzling that the sequence of intron 7 is not conserved at all across species: non-spliced intron 7 truncates human ERK1 while it increases the size of rat ERK1. On one hand, it is striking that the sequences of the correctly joined exons 7 and exon 8 are extremely conserved in all mammals, including the monotreme platypus which is phylogenically the most distant mammal to humans (Figure 2A). On the contrary, even when restricting the study to rodents, the protein sequences provided by intron 7 are absolutely not conserved. The total lack of conserved protein motifs renders the alternatively spliced ERK1s extremely unlikely to play a function in the cell (Figure 2B). In rodents, for example mouse ERK1b has a smaller molecular weight than mouse ERK1 (−0.67 kD) while rat ERK1b has a larger molecular weight than rat ERK1 (+2.66 kD). Finally, in transgenic mice that express only ERK1 cDNA (after disruption of all endogenous alleles of *erk1* and *erk2*), these splice variants do not exist, while mice live and reproduce normally (Frémin et al., 2015). Therefore, this alternative splices of ERK1 protein cannot provide a rationale for a distinct function between ERK1 and ERK2 proteins.

### Protein sequences

Protein sequences of ERK1 and ERK2 are 84% identical in a given mammal; human ERK1 is larger than human ERK2 due to an extension of 17 amino-acids at its N-terminal and 2 amino-acids at its C-terminal. The only described isoform-specific difference leading to functional difference resides in the N-terminal of mammalian ERK1. One report indicates that nuclear localization of ERK1 is slower than that of ERK2 due to the 20 amino-acids of ERK1 situated immediately after the poly-alanine stretch at N-terminal end (Marchi et al., 2008). However, no mechanism that could account for this difference has been reported so far, and again mice expressing only ERK1 are perfectly viable and fertile; hence this difference in rate of nuclear entry is not sufficient to block normal regulation of ERK signaling.

### Expression levels of ERKs

ERK2 is expressed at higher levels than ERK1 in most mammalian tissues (Busca et al., 2015; Frémin et al., 2015). One origin of this difference resides on the strength of the proximal promoters (1 kb upstream ATG codon), mouse *erk2* promoters being much stronger than mouse *erk1* promoter in transiently transfected NIH3T3 cells (Busca et al., 2015). However, the difference of strength between mouse *erk* promoters is larger than the steady state protein ratio measured (20% ERK1 and 80% ERK2, Lefloch et al., 2008); therefore further research is needed to understand the individual contribution of enhancers, RNA regulation and protein stability to establish the final ERK1/ERK2 protein ratio. One can note that mouse *erk1* RNA has only one short 3-prime UTR (632 bp) whereas mouse ERK2 RNA has a long 3-prime UTR (3777 bp) displaying also an alternative poly-adenylation site (transcripts mapk1-001 and -002 from Ensembl release 83). Does this long 3-prime UTR of ERK2 mouse mRNA provide additional regulation mechanisms? It is important to note that ERK expression is elevated, calculated to be in the μM range by several authors (reviewed in Fujioka et al., 2006). This concentration seems lower than that of MEK but markedly higher than that of Raf. More importantly, when proteomics measurements were performed systematically in mouse NIH3T3 cells, it was shown that ERK2 is among the 400 most expressed cell proteins (twice more expressed than PKA-catalytic subunit for example), and ERK1 is still among the 1500 most expressed cell proteins (10 fold more than p70-S6K for example; Schwanhausser et al., 2011). ERK protein expression is very stable and to our knowledge no stimulus-induced variations of protein quantities have been observed. The half-life of both ERK1 and ERK2 are very long, being of 68 and 53 h respectively as determined by proteomics analysis (Schwanhausser et al., 2011). ERK1 and ERK2 are expressed at different levels and apart from a clear difference in proximal promoter strength that was demonstrated recently, more work is needed to understand the regulation of ERKs protein expression.

### Structural changes upon activation

The crystal structure of ERK2 protein was the second kinase structure to be resolved after PKA catalytic subunit (Zhang et al., 1994), at present many studies have described the crystal structure of ERK1 and ERK2 when bound to partners and small pharmacological inhibitors. Dual phosphorylation of ERK triggers dramatic conformational changes within the activation lip, reorganizing the substrate binding site to enable recognition of the proline-directed phosphorylation motif of substrates (Pro-X-pSer/pThr-Pro), and reorienting active site residues involved in catalysis (Xiao et al., 2014). Side by side comparison of ERK1 and ERK2 3D structures highlights their close homologies (Ring et al., 2011). However, it was shown, by measuring hydrogen/deuterium exchange, that constraints at the hinge between the lobes of ERK2 were released during activation, which does not seem to be the case for ERK1 (Ring et al., 2011). The functional consequences of this difference have not been currently deciphered since both kinases dramatically increase their catalytic activity upon phosphorylation.

### Mechanism of ERKs activation and substrate recognition

ERK1 and ERK2 are simultaneously activated by the same external growth factor agonists, indeed *in vitro* purified MEK1/2 can phosphorylate indiscriminately ERK1 and ERK2 (Robbins et al., 1993). In fact close analysis of isoforms upstream in the cascade failed to identify isoform-specific signaling cascades (Lefloch et al., 2009). The ratio of active ERKs mimics exactly the ratio of ERK proteins expressed in the cell, furthermore when expression of one isoform is silenced, activation of the remaining isoform is increased. Collectively these observations indicate that MEK activates indistinctively ERK1 and ERK2 and that both compete each other for the upstream activating kinase (Lefloch et al., 2008). Both ERK1 and ERK2 were shown to translocate to the nucleus upon stimulation (Lenormand et al., 1993). Bacterially expressed ERK1 and ERK2 present similar specific activities *in vitro* (Robbins et al., 1993), and previous works of our group showed that this was also the case for immuno-precipitated epitope-tagged ERK1 and ERK2 from HeLa cells (Lefloch et al., 2008). These observations tend to indicate that ERK1 and ERK2 phosphorylate their substrates with the same efficiency, indeed it was shown that all the 284 interactors that bind to ERK2 also bind to ERK1 (von Kriegsheim et al., 2009). In addition, ERK1 and ERK2 share 22 out of 23 amino acids that have been demonstrated to directly interact with substrates (Lee et al., 2004; Liu et al., 2006), the sole difference being a conservative substitution: leucine^155ERK2^ into isoleucine^175ERK1^ (Busca et al., 2015). Therefore, if we consider these data on ERKs activation mechanisms and substrate recognition, no major differences seem to exist between ERK1 and ERK2.

### “Dimerization” domains

**Figure 3:**
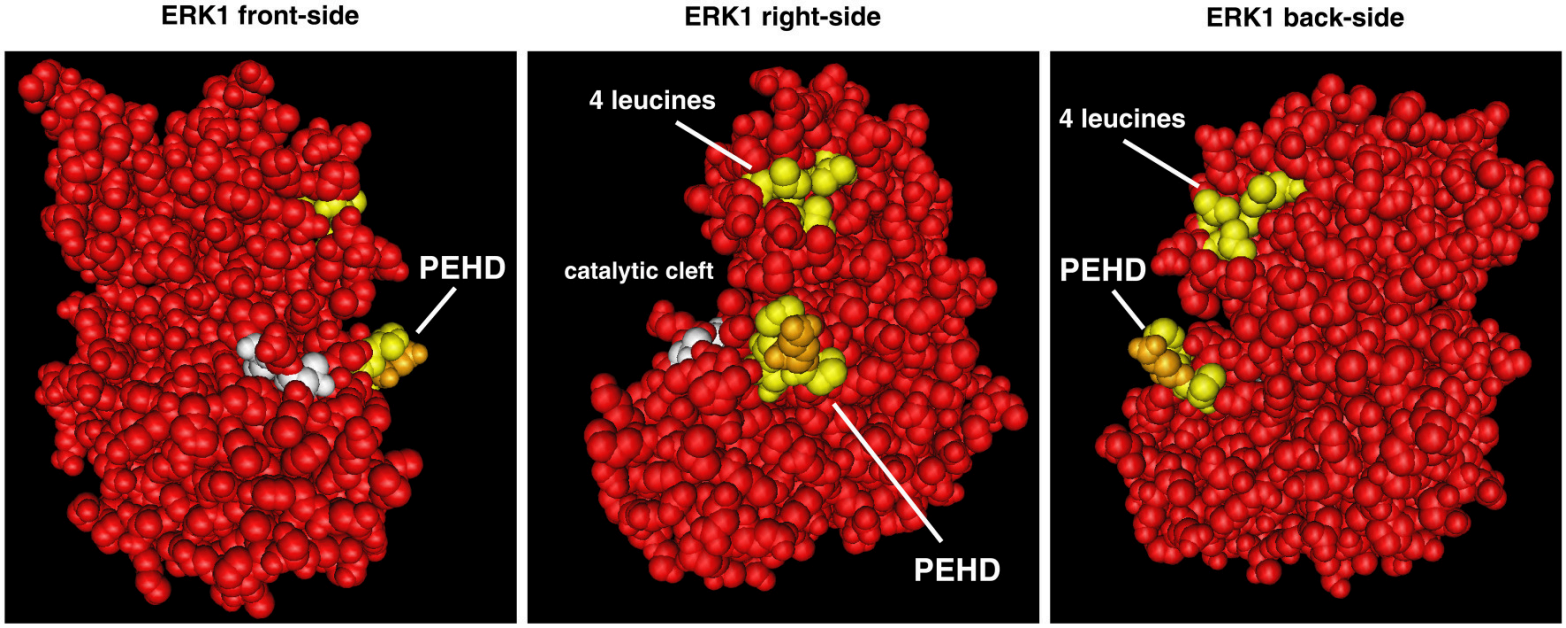
**Position of ERK “dimerization” domains on 3D representation of ERK1**. The two domains of ERK implicated in “dimerization” are highlighted in yellow on the 3D structure of ERK1 (still-images of structure 4QTB from RCSB PDB, www.rcsb.org, Chaikuad et al., 2014 viewed with CN3D software). “4 leucines” of ERK1 are equivalent to leucines L333, L336, L341, and L344 of mouse ERK2 (Khokhlatchev et al., 1998). In the sequence PEHD (equivalent to P^174^DHD^177^ of mouse ERK2), the glutamic acid of mammalian ERK1s is highlighted in gold. On mammalian ERK2s this glutamic-acid is replaced by an aspartic acid that is less bulky.

Two decades ago Khokhlatchev et al. identified surface residues that could interact to stabilize an ERK2-ERK2 dimer via studying ERK2 crystals (Khokhlatchev et al., 1998). To assist in the presentation of dimerization data in the literature we have drawn a 3D image of ERK1 (from Protein Data Bank (Berman et al., 2000) where the putative dimerization residues are highlighted in yellow and gold (Figure 3) on structure 4QTB (Chaikuad et al., 2014). These residues are located on the back of the kinase with a group of four leucines and the sequence PE/DHD that generates a protruding structure. ERK dimerization studies have already been extensively reviewed (Lee and Bae, 2012; Roskoski, 2012). Here we will summarize our current understanding of this process with an emphasis on differences among mammalian ERK1s and ERK2s.

Several studies have failed to observe dimerization of ERK molecules in cells. For example, studies from our group using real time fluorescence microscopy and fluorescence correlation spectroscopy failed to detect any dimer formation (Lidke et al., 2010). Similarly, Burack and Shaw failed to demonstrate ERK2 dimerization in live cells through FRET measurements between co-expressed yellow and cyan fluorescent ERK2s (Burack and Shaw, 2005). By using gel filtration chromatography coupled with multi-angle laser light scattering, Callaway et al. (2006) revealed that histidine-tagged ERK2 is overwhelmingly present as a monomer. Finally with an array of biochemical means, Kaoud et al. have shown that ERK2 without any tag was strictly monomeric, whereas only His-tagged ERK2 could partially form a dimer (Kaoud et al., 2011). More recently, Herrero et al. have reported that ERK2 from chicken embryo extracts does not form dimers unlike ERK2 from mouse cells. Since ERK2s from these two animals are over 99% identical (outside the alanine-rich N-term that is highly variable among all ERKs) and share the same “ERK-dimerization” domains it confirms that complex formation depends on the cellular context (partners) but it does not depend on the ERK2 protein itself (dimer formation).

The lack of ERK dimerization from these studies mentioned above does not invalidate the initial observation of complex formation and dimerization-mutants have proven to be very instructive. For example we showed that dimer^mutant^-ERK1 activation by MEK and its nuclear entry were delayed (Lidke et al., 2010). Vomastek and co-workers have shown that dimer^mutant^-ERK2 failed to associate with the Trp protein, a component of the nuclear pore (Vomastek et al., 2008). They further demonstrated that Trp-ERK2 association did not use classical ERK docking sites. More recently, Herrero et al. have shown that ERK's ability to form “dimers” was correlated to PEA15 expression level (Herrero et al., 2015); PEA15 is a well-known ERK partner (Formstecher et al., 2001). Even more interestingly, they have identified a small molecule inhibitor that blocks “dimerization” (it blocks the slower migration of stimulated ERK in native PAGE electrophoresis; Herrero et al., 2015). This inhibitor specifically blocked activation of cytoplasmic ERK substrates and impeded tumorigenesis driven by oncogenes of the RAS/ERK pathway and is the first of a new class of inhibitors that targets interactions of ERK with partners instead of blocking ERK activity. This inhibitor should also help to understand the exact nature of the interaction between ERKs and partner(s) via these “dimerization domains” (Herrero et al., 2015).

Regarding differences between ERK isoforms, the four leucines of the “dimerization” domain are conserved among ERKs of all tetrapods. Therefore, they cannot provide grounds for differences between ERK1 and ERK2. However, all mammalian ERK1s share the PEHD sequence, whereas all mammalian ERK2s have the sequence PDHD (the glutamic acid of ERK1 (E) is highlighted in gold in Figure 3). Changing glutamic-acid for aspartic-acid is considered a conserved substitution due to their overall negative charge in solution, therefore via their PEHD or PDHD sequences, ERK1s and ERK2s should bind similarly to interactors. Nonetheless, glutamic acid is bulkier since it has two carbon atoms on its side chain instead of one for aspartic-acid. Therefore, mammalian ERK1s may display a more restricted pattern of interactions with partners by requiring a deeper pocket since these sequences generate protruding structures (Figure 3). Interestingly, among all tetrapods, only ERK1s from squamates (snakes, lizards, and geckos) have the aspartic acid at this position. As we shall see later, these animals express only ERK1, which could indicate that the PDHD sequence is more universal than PEHD, being the sequence of tetrapods that express only ERK1 (squamates) and all tetrapods'ERK2s, including animals expressing only ERK2 (birds and frogs). However, invertebrates have only one ancestral ERK that present either glutamic or aspartic acid at this position, and even at the base of vertebrates, lamprey's only ERK has an aspartic acid whereas hagfish unique ERK has a glutamic acid. Therefore, the structural difference, PEHD in mammalian ERK1s vs. PDHD in mammalian ERK2s, is likely to have minor consequences on interaction with partners.

Although, many studies have failed to show ERK dimerization, the two “dimerization domains” play a role in ERK response. The minor structural differences between ERK1 and ERK2 in the PE/DHD domain may trigger minor signaling differences that may be uncovered when partners that assemble with ERK via this domain will be fully identified.

## Search for specific functions of ERK isoforms

Upstream of ERK in the signaling cascade, clear functional differences among isoforms have been demonstrated. For example, at the Raf level, B-Raf displays a greater specific activity than A-Raf and C-Raf, and partners of Raf kinases vary greatly (Desideri et al., 2015). Indeed B-Raf is already primed for MEK activation via constitutive phosphorylation of Ser^445^ and amino-acids negatively charged that mimic the phosphorylation status observed in activated A-Raf or C-Raf isoforms (Asp^448^ in B-Raf) (Tran et al., 2005). At the level of MEK1 and MEK2, only MEK1 can be regulated by multiple phosphorylation of its proline-rich domain. MEK1 can be retro-phosphorylated by ERK (Saito et al., 1994) in the context of phosphorylation by PAK kinase that transmits signaling from the cell matrix (Eblen et al., 2004).

Clear differences in the pattern of tissue-specific expression among isoforms as also been demonstrated upstream of ERK. For example, B-Raf has been described to be more expressed in cells of neuronal origin (Storm et al., 1990) and MEK2 was shown to be preferentially expressed in embryos and excluded from adult brains (Alessandrini et al., 1997; Di Benedetto et al., 2007). Taken together, all these observations indicate that the role of individual isoforms can be unique in the pathway; therefore the quest to understand putative functional differences between ERK1 and ERK2 is relevant to further understand all regulatory aspects of this signaling cascade.

In order to seek for specific ERK1 and ERK2 functions, their respective expression levels have been reduced by distinct means and phenotypical consequences have been analyzed. Expression of ERK1 and/or ERK2 has been silenced *via* expression of morpholinos in zebrafish, shRNA/siRNAs in cultured cells or disrupted by gene knock-out in mice. Since ERK1 and ERK2 proteins are usually not expressed at the same level, it is very important to take into account the relative level of both proteins to interpret the data. We want to emphasize that only the dual phosphorylated and activated forms of ERK1 and ERK2 define a common conserved epitope recognized by the same anti-phospho antibody (Busca et al., 2015). Indeed, the direct relationship between the ratio of phosphorylated ERKs and the quantitative expression ratio of ERKs has been previously shown (Lefloch et al., 2008), therefore the easiest way to evaluate the ratio between ERK1 and ERK2 in a biological sample consists in measuring the ratio between the dually-phosphorylated ERK isoforms. Ideally it would be of interest to add-back either isoform by recombinant vectors; unfortunately it is difficult to express successfully ERK cDNAs that can be activated as efficiently as the endogenous ERK. Usually a smaller proportion of transfected ERK is activated compared to endogenous protein (one example is found in Figure 6 of Radtke et al., 2013).

### Morpholinos

Morpholinos (modified antisense oligo-nucleotides that are biologically stable) have been injected into zebrafish embryos to assess the contributions of ERK1 and ERK2 during zebrafish development. From the same laboratory, one study concluded that ERK1 and ERK2 target common and distinct gene sets during embryogenesis (Krens et al., 2008a) while another study concluded that cell migration defects during gastrulation were more pronounced upon ERK2 knock-down (Krens et al., 2008b). In this latter publication the authors indicate that morpholinos-mediated knock-down of ERKs could be rescued by co-injection of the corresponding mRNA. Strikingly, *erk2* mRNA cross-rescued ERK1 knockdown, but *erk1* mRNA was unable to rescue ERK2 knockdown. These results tend to indicate that ERK1 and ERK2 play different roles in zebrafish development. A close look of the data reveals that ERK2 morpholinos reduce markedly more the global level of phospho-ERK than ERK1 morpholinos (measured by immuno-histochemistry with anti-phospho ERK antibody at 4.5 and 8 h post fertilization, Figure 6 in Krens et al., 2008b). Furthermore, when evaluating the expression level of six genes at 4.5 h post fertilization (goosecoid; antivin; vox; vent; notail; tbx6), injection of morpholinos targeting ERK1 reduced mildly the level of 5 genes, whereas morpholinos targeting ERK2 reduced strongly the level of all genes (Figure 10 in Krens et al., 2008a). Taken together, these two observations seem to indicate that ERK2 is more expressed than ERK1 in zebrafish embryos, consequently the different outcome observed when reducing ERK1 or ERK2 quantity could be linked to their different effectiveness to reduce global ERK activity, not due to isoform-specific functions. Injection of *erk1* RNA did not rescue anti-ERK2 morpholinos, whereas the opposite situation was effective. However, in this study no data indicate that *erk1* RNA re-established a normal level of global phospho-ERKs to conclude unambiguously an ERK1-specific effect.

### Silencing by siRNA and shRNAs

Knock-down of ERK isoforms using specific siRNAs has been used in several studies to replace chemical inhibitors that inhibit ERK activity in cells. For example, in two lines of ovarian-cancers cells (HeyC2 and KGN), simultaneous diminution of ERK1 and ERK2 by a pool of siRNA was shown to reduce cell proliferation more effectively than MEK inhibition by either one of two chemical inhibitors (PD98059 and U0126; Steinmetz et al., 2004).

At least 27 publications have evaluated the role of individual ERK isoforms in biological processes via siRNA mediated knock-downs, and 4 more publications have combined the expression of shRNA directed to ERK2 with genomic *erk1* gene knock-out. It must be noted that removal of ERK1 alone induced phenotypical changes in 13 studies (Table 1); we can therefore conclude that ERK1 plays significant roles in many mammalian tissues that can be compensated in ERK1^−∕−^ animals. The fact that ERK1 has a functional role in mammals can also be deduced from the high stability of ERK1 protein sequence during evolution (Busca et al., 2015), if ERK1 had been dispensable its sequence would have derived rapidly.

**Table 1:** **Overview of studies using shRNA or siRNA transfections to study ERK1 vs. ERK2 signaling**.

**References**	**ERK1/2 ratio**	**Effect on phospho-ERKs**	**Cell line**	**Phenotypes studied**
**PHENOTYPES ONLY ERK1-DEPENDENT**				
Zhong et al., 2009	E1 ≥ E2	Yes	Rat hepatic stellate HSC-T6	Cell proliferation, gene induction, hepatic fibrosis
Jung et al., 2012	E2 ≥ E1	Not done	Rat vascular smooth muscle cells	Cordycepin dependant block of cell proliferation
Bae et al., 2013	E2 > E1	Not done	Human pulmonary NCI-H292	IGF1-dependant MUC8 and MUC5B induction
**PHENOTYPES ONLY ERK2-DEPENDENT**				
Vantaggiato et al., 2006	E2 > E1	yes	Mouse embryo fibroblasts + NIH3T3	Cell proliferation, colony and tumor formation
Li and Johnson, 2006	E2 > E1	Yes	Mouse myoblasts C2C12	Myoblast proliferation and differentiation
Wille et al., 2007	E2 > E1	Yes	Hybridoma 1B6 T	IL-2 production from TCR stimulation
Bessard et al., 2008	E2 > E1	Yes	Rat hepatoma cell line, rat biliary epithel.	Hepatocytes proliferation
Carcamo-Orive et al., 2008	E2 > E1	Not done	Human mesenchymal stem cells	Proliferation and adipogenic differentiation
Li et al., 2009	E2 > E1	Yes	Mouse NIH3T3	TGF-beta1-induced collagen synthesis
Shin et al., 2010	E2 > E1	Not done	Human mammary gland MCF-10A	Epithelial-to-mesenchymal transformation
Botta et al., 2012	Not done	Not done	H. pancreatic ductal epithelial cells	Cell invasion, MMP RNA increase
Lee et al., 2013	E2 > E1	Yes	Human HSC-3 and MDA-MB-231	Expression of tumor-derived G-CSF
Radtke et al., 2013	E2 > E1	Yes	H. non-small cell lung carcinoma A549	HGF-induced cell motility, paxillin phosphorylation
Shin et al., 2013	E2 > E1	Yes	Mouse embryo fibroblasts	Increase p19^mArf^ and p16^Ink4a^, senescence
Bonito et al., 2014	E2 > E1	Yes	Human osteosarcoma cells U2OS	Expression of cytokine receptor sub-unit gp130
Gusenbauer et al., 2015	E2 > E1	Not done	H. squamous carcinoma cell SCC9	Amphiregulin upregulation by HGF
Chang et al., 2015	E2 > E1	Not done	H. monocytic leukemia cell line THP-1	LPS-induced G-CSF
**PHENOTYPES ERK1 and ERK2-DEPENDENT**				
Zeng et al., 2005	E2 > E1	Yes	Human ovarian epithelium pOSE	Cell viability
Lefloch et al., 2008	E2 > E1	Yes	Mouse NIH3T3	Cell proliferation
Wei et al., 2010	Not done	Not done	Human breast MCF7	Etoposide-induced G2/M arrest, ATM pathway
Wang et al., 2011	E2 > E1	Yes	Human chondrocytes	Osteoarthritis/cartilage breakdown
Wei et al., 2011	E1 = E2	Not done	Human breast MCF7	Hydroxy-urea induced DNA damage response
Shukla et al., 2011	Not done	Not done	Human mesotheliomas HMESO	Cell proliferation, migration and tumor growth
Woodson and Kedes, 2012	E2 > E1	Yes	Rhesus monkey fibroblasts	RRV virus production and localization inside virion
Qin et al., 2012	E2 > E1	Yes	Human A375 melanoma cells	Cell proliferation and cell death
Frémin et al., 2012	E2 ≥ E1	Yes	Rat hepatocytes	Survival, proliferation, differentiation state
Zhu et al., 2015	E2>E1	Yes	H. rhabdomyosarcoma RD	Enterovirus (EV71) replication
**shRNA MEDIATED SILENCINGS AND** ***erk1*** **GENE DISRUPTION**				
Fremin et al., 2007	E1 = E2	Yes	Primary murine hepatocytes	Cell proliferation
Frémin et al., 2009	E2 ≥ E1	Yes	Primary rat hepatocytes	Cell proliferation and survival
Voisin et al., 2010	E1 ≥ E2	Yes	Mouse embryo fibroblasts	Cell proliferation
Guegan et al., 2013	E2 > E1	Yes	H. hepatocellular carcinoma cells Huh-7	Cisplatin-induced cell death

“*ERK1/2 ratio” (2nd column) was determined indirectly from western-blots probed with anti-phospho-ERK antibodies revealing active ERK levels, as demonstrated previously (Lefloch et al., 2008). “Effect on phospho-ERKs” (3rd column) indicates whether or not the impact of shRNAs or siRNAs on phospho-ERKs levels was evaluated by western-blot. Only the main phenotypes studied are presented (last column)*.

#### ERK1-specific effects

Two studies have concluded that reduction of ERK1 expression was effective to induce phenotypes whereas reducing ERK2 expression was ineffective. In the study of Jung et al. the authors tested the role of ERK isoforms on the action of cordycepin on vascular smooth muscle cells (VSMC) (Jung et al., 2012). In VSMC, cordycepin increases the levels of p27KIP1 while it reduces both CDK4 levels and cell proliferation. These three actions of cordycepin were reversed by transfection of a single siRNA targeting ERK1 but not by a siRNA targeting ERK2. In these cells, the expression levels of ERK1 and ERK2 appeared very similar, or slightly higher for ERK2, if we look at the presented phospho-ERK immuno-blots. Unfortunately, the effectiveness of each siRNA to reduce the protein level of its target was not shown; hence it is not possible to correlate the decrease of global active ERK by the siRNAs with the phenotypical consequences. In the study of Bae et al. (2013), a single siRNA was also used against each ERK, and the effectiveness of each siRNAs to reduce ERKs levels was not presented, nor was shown the effectiveness of each siRNA to reduce the global active ERK levels. The authors claim that only transfection of siRNA targeting ERK1 reduced the IGF-1 mediated induction of two mucin genes, in NCIH292 airway epithelial cells (Bae et al., 2013). Altogether, the lack of controls in these two publications impairs concluding that only the decrease of ERK1 expression can trigger specific phenotypes (Jung et al., 2012; Bae et al., 2013).

#### Different effects upon silencing ERK1 or ERK2

Three publications propose different outcomes after knocking-down of ERK1 vs. ERK2 (one that will be discussed in the following paragraph on hepatocytes). In rhesus fibroblasts infected with rhadinovirus, Woodson and Kedes. conclude that knock-down of ERK1 seems to increase viral production whereas ERK2 accumulates preferentially into viral particles (respectively Figures 9, 10 in Woodson and Kedes, 2012). In these cells ERK2 is much more expressed than ERK1 as determined by phospho-ERK levels (Figure 6A of Woodson and Kedes, 2012) and in reality ERK2 does not accumulates preferentially in virions since the authors state in the caption of their Figure 8 that “intravirion ERK content reflects intracellular expression of ERK isoforms”. Further, of concerns for interpreting the data in Figure 8 of Woodson and Kedes (2012), the total-ERK and phospho-ERK blots have been inverted.

In a study regarding human epithelioid malignant mesotheliomas (Shukla et al., 2011), the authors propose that ERK1 and ERK2 play different functions on the ground that gene expression is altered differently in stable clones expressing less ERK1 vs. stable clones expressing less ERK2. Unfortunately one cannot find measurement of the relative level of ERK1 and ERK2 since no phospho-specific antibodies were used, and this makes the interpretation of the data very difficult. Could their results be simply due to different contributions of ERK1 and ERK2 to global ERK activity? (Shukla et al., 2011).

#### ERK2-specific effects

##### In hepatocytes

Fourteen publications state that ERK2 silencing triggers biological consequences whereas ERK1 silencing does not. Among these studies, several were performed in hepatocytes with contrasting results that we have attempted to analyze. At first glance, in most experiments hepatocytes seem to express equivalent levels of ERK1 and ERK2, an ideal situation to compare the contribution of these isoforms to conduct ERK signaling. However, a closer look at the data reveals that it is clearly not the case in all hepatocytes cultures; for example in hepatocytes from a publication of Figure 3 in Frémin et al. (2009) there is clearly much more ERK2 than ERK1 (similarly in stellar HSC-T6 cells from the study of Figure 1C in Zhong et al., 2009). In a single publication, the ERK1/ERK2 ratio seems to vary between experiments and the reason for these changes is not explained. One explanation could be the very low level of active ERKs in most of the hepatocytes preparation, a low level that magnifies the background from antibodies that have some weak affinity for mono or even non-phosphorylated forms of ERKs. This situation has been encountered by Jung et al. in vascular smooth muscle cells, in cells where global ERK activity is elevated the immunoblot reveals much more phospho-ERK2 than phospho-ERK1 (Figure 4A in Jung et al., 2012); whereas there was not much difference between phospho-ERK1/ERK2 in the blot of their Figure 4B when ERK activity was much lower. The same conclusion can be drawn from Figure 1A in a study from Steinmetz et al. (2004), where extracts from the same cell line are loaded side by side, only the extracts with high level of phospho-ERK demonstrate the clear prevalence of ERK2 (Steinmetz et al., 2004).

Furthermore, in hepatocytes it was shown for the first time that following ERK1 or ERK2 decrease, the remaining isoform was over-activated up to 11 fold (Fremin et al., 2007). Therefore, the global strength of ERK activation following removal of ERK1 or ERK2 needs to be measured, which is usually difficult to do when the activation levels are low, as it seems to be the case in most hepatocytes preparations. A clear example of this problem is shown in Figure 6D from a study by Frémin et al. (2012) where global phospho ERK seemed to be increased upon knock-down of ERK1 due to increased intensity of phospho-ERK2 band. Indeed, opposite consequences on phenotypes have been found; for example Zhong et al. (2009) have shown that decrease of ERK1 suppressed hepatic fibrosis and reduced cell proliferation. On the contrary Frémin et al. have concluded that only ERK2 silencing reduced hepatocytes proliferation (Fremin et al., 2007). In a later study, these authors have shown that ERK1 silencing could enhance hepatocyte survival (Frémin et al., 2009). More recently it has been shown that dual silencing of both ERK1 and ERK2 is required to maintain a highly differentiated state of hepatocytes, while survival and proliferation was suggested to be regulated via a complex interplay between ERK1 and ERK2 functions (Frémin et al., 2012). Similarly the fate of PC12 cells toward proliferation or differentiation was shown to be dependent on the strength and duration of ERK signaling, therefore a precise knowledge of the global level of active ERK is required all along these long lasting processes, to be able to effectively assign a function to ERK1 vs. ERK2 isoforms (Dikic et al., 1994; Traverse et al., 1994). Even small differences in ERK1/ERK2 expression level could be translated into differences of global ERK level, leading into different phenotypical consequences.

##### ERK2-specific effects in other cell lines

Twelve studies, independent of those using hepatocytes, have revealed phenotypes induced only by ERK2 decrease, it is essential to note that in 11 of them, cells express markedly more ERK2 which by itself could provide an explanation for the lack of effect of ERK1 knock down as demonstrated previously in NIH3T3 cells (Lefloch et al., 2008). In the last study out, the ratio cannot be determined by lack of phospho-ERK immunoblot (Botta et al., 2012).

In the studies of Chang et al. (2015), Gusenbauer et al. (2015), and Carcamo-Orive et al. (2008) unfortunately the effectiveness of siRNAs to reduce global ERK activation is not demonstrated, impeding to draw conclusions. Bonito et al. (2014) report that differences between both ERKs knock-down could not be attributed to quantitative differences because they extend their work to MCF7 cells which they claim to express the same level of ERK1 and ERK2. This remains to be demonstrated unambiguously, since unfortunately they did not measure phospho-ERK levels in those cells; furthermore the effect of ERK2 silencing triggered only a 30–40% diminution of GP130 expression, therefore a 10–20% decrease that could be caused by ERK1 silencing (if ERK1 was slightly less expressed than ERK2), would be difficult to demonstrate. Most of their conclusions were drawn from cell lines expressing markedly more ERK2 than ERK1 (Bonito et al., 2014). Finally, the studies of Li and Johnson (2006), Shin et al. (2013), and Li et al. (2009) have shown that only ERK2 silencing markedly reduces global ERK activity, providing a direct explanation for the lack of consequences after ERK1 knock-down.

##### Studies proposing ERK2-specific roles from add-back strategies

In MCF-10A breast cancer cells, Shin and co-workers elegantly demonstrate that the epithelium-mesenchymal transition is dependent on ERK activity via interaction with substrates containing DEF docking sites (Shin et al., 2010). However, these authors did not assess the effectiveness of the shRNAs to decrease global ERK activity, nor did they present the capacity of transfected ERK isoforms to re-establish the global ERK activity after knock-down. Direct comparison of ERK1 vs. ERK2 expression from transfected plasmids is rendered complicated by the use of different tags in ERK1 and ERK2 (measurement of expression with total ERK antibody could introduce an isoform-bias as discussed above). Furthermore, Figure S4 in Shin et al. (2010) reveals that ERK2 is much more expressed than ERK1 in those cells, which could explain why only ERK2 silencing impeded epithelio-mesenchymal transition. Vantaggiato and co-workers have also transfected ERK isoforms and showed that ERK1 expression was able to block Ras-induced increase of colony formation in contrast to transfected ERK2, however the size of transfected ERK1 is abnormally smaller than that of ERK2, rendering difficult the interpretation of the results (Vantaggiato et al., 2006). These authors have also shown that removal of ERK1 in a stable line of mouse embryo fibroblasts (MEFs) accelerates the rate of cell proliferation whereas removal of ERK2 decreases this parameter. This observation is surprising, first because the ERK1 removal in MEFs was demonstrated by others to slow cell proliferation (in five MEFs preparations of early cell culture passages; Voisin et al., 2010), and second because mice over-expressing ERK1 live and reproduce normally, even in the total absence of ERK2 protein (Frémin et al., 2015). Indeed Voisin et al. have demonstrated that individual loss of either ERK1 or ERK2 slows down the proliferation rate of fibroblasts to an extent reflecting the expression level of the kinases (Voisin et al., 2010). In A549 lung carcinoma cells, Radtke et al. (2013) concluded that only ERK2 mediates HGF-induced motility. In these cells ERK2 is markedly more expressed than ERK1, and in Figure 5A of the paper only shERK2 is shown to reduce global ERK activity which could explain why only an ERK2-mediated effect was observed. In an add-back experiment (Figure 6 in Radtke et al., 2013) HA-tagged ERK proteins are hugely expressed and furthermore only transfected HA-ERK2 was markedly stimulated upon HGF treatment. This can explain again why only ERK2 mediates the action of HGF (Radtke et al., 2013).

##### One well controlled study reporting ERK2-specific effect

In HSC-3 cells, Lee et al. report that a decrease in ERK1 expression reduces more effectively global ERK activity than ERK2 decrease, but only ERK2 knockdown reduces G-CSF mRNA levels (Figure 5 in Lee et al., 2013). For the first time, this study clearly favors a distinct role for ERK isoforms, however in the same study with another cell line (MDA-MB-231) the same authors report a direct correlation between the global ERK activation (reduced significantly only by ERK2 knockdown) and G-CSF mRNA levels.

#### Redundant effects

If we consider the remaining 10 studies that report a biological consequence after reducing ERK1 quantity, all of them have also revealed a specific phenotype upon ERK2 protein diminution. Most of these studies (7 out of 10) reveal a redundant role of both ERK isoforms. Unfortunately, for the study of Zeng et al. (2005) (siRNAs in cancerous ovarian cells) and for two publications from the group of D. Tang (siRNA in MCF7 cells, Wei et al., 2010, 2011) the decrease in phospho-ERKs was not measured, rendering impossible to correlate the higher effectiveness of ERK1 knock-down to a slower ovarian cell proliferation for example. Surprisingly the same blot was used in both papers from the group of D. Tang to illustrate the effectiveness of each shRNA to reduce expression of their targets.

Fortunately several publications have presented the effectiveness of knock-downs to regulate global ERK activity, allowing to correctly interpreting the data. For example, the production of EV71 viral particles in Rhabdomyoscarcoma cells was shown to be equally diminished by reduction of ERK1 or ERK2 expression (Zhu et al., 2015). Here, both siRNAs effectively reduced the global level of active ERKs, despite the fact that these cells seem to express more ERK2. Pharmacological inhibition of MEK activity by U0126 inhibitor was more effective to reduce global ERK phosphorylation level, and was shown to further reduce the production of viral particles (Zhu et al., 2015). In human melanoma cell line A375, Qin et al. have shown that silencing of ERK1 or ERK2 reduces the levels of active ERK and killed the cells by similar apoptosis induction. Silencing of ERK isoform was performed with lentiviral particles that express shRNAs. Interestingly enough, ERK1 silencing was sufficient to reduce the global levels of active ERK, although the expression level of ERK1 is somewhat lower than that of ERK2 in these cells (Qin et al., 2012). In human chondrocytes infected with lentivirus expressing shRNA, Wang et al. (2011) showed that knock-down of either ERK1 orERK2 reduces the mRNA levels of MMP3 and MMP13 and type II collagen, while double knock-down of ERK1 and ERK2 acted synergistically. Furthermore, there is a correlation between the effectiveness of the shRNAs to reduce the global levels of active ERK and the consequences on MMPs and collagen II expression.

#### Conclusions from studies with siRNAs and shRNAs

Overall nearly all publications claiming a specific role of ERK2 can be re-interpreted by observing that higher reduction of global ERK activity upon ERK2 silencing induces more effects than upon ERK1 silencing. Doubts persist only in studies that cannot be carefully interpreted by lack of assessment of the contribution of each isoform to the global ERK activity. Only in one out of two cell lines of the study of Lee et al. (2013) was demonstrated an ERK2-specific effect. In the few cell lines studied that express more ERK1 than ERK2, ERK1 was demonstrated to be the pre-eminent isoform driving defined phenotypes. Therefore, in all but one study, the observed phenotype develops proportionally to the reduction of the global ERK activity.

Of special interest is the work of Wille et al. who used stable expression of shRNA targeting ERK1 or ERK2. The stable clones generated expressed various levels of shRNAs, producing a quantitative range of knock-downs in mouse 1B6 hybridoma cells (Wille et al., 2007). These authors demonstrate a strict correlation between global ERK activity and a signal output (IL-2 production) irrespective of the targeted isoform.

Similarly, in NIH3T3 it has been demonstrated that ERK1 knock-down decreases cell proliferation only when ERK2 is lowered at the same time to a threshold level. In these NIH3T3 fibroblasts, ERK activity is mainly provided by the ERK2 isoform that represents 80% of the total ERK pool (Lefloch et al., 2008). Globally, experiments knocking-down ERK1 and ERK2 by siRNAs and shRNAs converge to reveal that ERK1 and ERK2 contribute to ERK signaling according to their contribution to global ERK activation; therefore suggesting their functional redundancy at least for the read-out reported.

### *erk1, erk2* gene disruption

Several studies have tried to uncover functional differences between ERK1 and ERK2 by gene knock out in mice. Mouse ERK1 was disrupted by removal of exon 3 (Pages et al., 1999). ERK1^−∕−^ animals lived and reproduced normally in striking contrast to *erk2* disruption that led to early embryonic death (Hatano et al., 2003; Saba-El-Leil et al., 2003; Yao et al., 2003). The opposite fates of ERK1 vs. ERK2 invalidations initiated the quest to discover whether these two conserved kinases play distinct roles. Lack of ERK2 led to placental failure that could account for the early lethality (Hatano et al., 2003; Saba-El-Leil et al., 2003). When the placental defect was rescued by tetraploid-aggregation, ERK2-deficient fetus grew as well as littermate controls for 5 more days, up to E13.5 (Hatano et al., 2003), however this rescue did not allow animals to be born alive. To remove the possibility that lethality was due to a delayed placental failure, the group of Meloche has recently shown that *erk2* disruption in the epiblast still led to lethality (they induced a CRE-dependent ERK2 knock-out in the whole embryo except placenta; Frémin et al., 2015). Indeed in a different mouse genetic background, it was shown that *erk2* disruption did not form mesoderm (Yao et al., 2003). Overall, ERK2 is absolutely required for mammal life at several stages of development. In adult mice already lacking ERK1, inducible invalidation of ERK2 led to death by multiple organ failures within weeks (Blasco et al., 2011).

In order to correctly interpret the data from genomic disruptions of ERK isoforms, one should at least analyze the contribution of ERK1 and ERK2 to the global ERK activity in wild-type tissues. Ideally total ERK activity of tissues from wild-type animals should be compared with tissues from mice lacking one ERK isoform, since lack of one isoform is partially compensated by over-activation of the remaining one.

#### *erk1* disruption

At least 17 studies describe consequences of ERK1 sole removal in mice. ERK1 being less expressed than ERK2 in most mouse tissues (Frémin et al., 2015), phenotypes are usually not dramatic and great care needs to be taken to interpret the data. For example it was initially reported that lack of ERK1 impeded terminal differentiation of CD4CD8 thymocytes (Pages et al., 1999), however studies with congenic mice (different genotypes in the same litter) led to conclude that thymocytes proliferate and differentiate normally in mice lacking ERK1 (Fischer et al., 2005; Nekrasova et al., 2005). Goplen and co-workers have shown by western-blot analysis that the lack of ERK1 decreased by half the global ERK activity in airway tissues thereby impairing mice lacking ERK1 to develop airway inflammation and hyper-reactivity to experimental asthma (Goplen et al., 2012). Table 2 illustrates the multiple phenotypes observed in mice lacking ERK1, such as resistance to obesity (Bost et al., 2005), increase in long-term memory (Mazzucchelli et al., 2002) and hyper-activity in open field (Selcher et al., 2001). Considering these multiple studies, although mice lacking ERK1 live and reproduce normally, ERK1 is an essential kinase which role is can be revealed upon challenging the ERK pathway. For example, mice lacking ERK1 develop less skin papilloma generated by DMBA and TPA treatment than wild-type mice (Bourcier et al., 2006).

**Table 2:** **Overview of studies using genomic disruption to study ERK1 vs. ERK2 signaling**.

**Refernces**	**ERK1/2 ratio**	**Effect on phospho-ERKs**	**Promoter of Cre recombinase**	**Main phenotypes**
***erk1*** **GENE DISRUPTION IN WHOLE MICE**				
Pages et al., 1999	E2 > E1	Yes		Thymocytes differentiation
Selcher et al., 2001	E1 = E2	Yes		Behavior, activity in the open field, fear, learning, fear acquisition
Mazzucchelli et al., 2002	E2 > E1	Yes		Long-term memory, rewarding properties of morphine
Nekrasova et al., 2005	Variable	Yes		Thymocytes differenciation, priming encephalomyelitis
Bost et al., 2005	E1 = E2	Not done		Adipose tissue development, obesity and insulin resistance
Agrawal et al., 2006	not done	Not done		Thymocytes Th1 polarization, immune response, encephalomyelitis
Ferguson et al., 2006	Not done	Not done		Psychomotor sensitization to cocaine, behavioral plasticity
Cestari et al., 2006	Not done	Not done		Memory reconsolidation, fear conditioning
Bourcier et al., 2006	E2 > E1	Yes/No		Cutaneous lesions, TPA+DMBA induction of skin papillomas
Nakazawa et al., 2008	E2 > E1	Yes		NMDA-induced retinal injury
Alter et al., 2010	E2 > E1	Yes		Formalin-induced long-term heat hypersensitivity, pain models
Lee et al., 2010	E2 > E1	Yes		Adiposity and adipogenesis, insulin resistance
Cisse et al., 2011	Not done	Not done		Secretion of N1 fragment of cellular prion protein PrP(c)
Jager et al., 2011	Not done	Not done		Obesity, insulin resistance, liver steatosis, glucose uptake
Saulnier et al., 2012	Not done	Not done		Osteopetrosis, differentiation of hematopoietic stem cells
Goplen et al., 2012	E1 = E2	Yes		Thymocytes Th2 differentiation, asthma
***erk2*** **GENE DISRUPTION IN WHOLE MICE**				
Saba-El-Leil et al., 2003				Embryonic lethality, placenta development
Yao et al., 2003				Embryonic lethality, mesoderm differentiation
Hatano et al., 2003				Embryonic lethality, placenta development
Lips et al., 2004	Not done	Yes (IP)		(Loss of only one erk2 allele) ischemia-reperfusion injury, infarction
Purcell et al., 2007	E2 > E1	Yes		(Loss of only one erk2 allele) cardiac hypertrophic growth response
***erk1*** **AND** ***erk2*** **DISRUPTION IN CELLS**				
Chen H. et al., 2015	E2 > E1	Not done		Self-renewal, genome stability and pluripotency of mouse ESCs
***erk2*** **GENE DISRUPTION IN TISSUES**				
Satoh et al., 2007	Not done	Not done	EIIA–Cre + partial ERK2	Long term memory, fear conditioning
Newbern et al., 2008	Not done	Not done	Wnt1:Cre (neural crest)	Developmental defects
Samuels et al., 2008	Not done	Not done	hGFAP–(neural progenitor)	Proliferation, differentiation, cognition, memory formation
Satoh et al., 2009	E2 = E1	Yes	EIIA-Cre	Re-epithelization, burn healing, keratinocytes proliferation
Hamilton et al., 2013	E2 > E1	Yes	Get embryonic stem cells	Pluripotency-associated transcripts
Ulm et al., 2014	On 2 gels	Not clear	MLC2v-CRE (cardiomyocyte)	Hypertrophic remodeling of cardiomyocytes, apoptosis
Frémin et al., 2015	E2 > E1	Yes	Sox2:Cre (whole embryo)	Embryo development with normal placenta
***erk2*** **GENE DISRUPTION IN TISSUES IN MICE LACKING** ***erk1*** **GENE**				
Fischer et al., 2005	Not done	Not done	Cd4-Cre and Lck-Cre	CD4 and CD8 T-cell lineage commitment
Ishii et al., 2012	Not done	Not done	CNP-Cre	Myelin growth, oligodendrocyte differentiation
Fan et al., 2009	E2 > E1	Yes	Cyp19-Cre	Oocyte activation, ovulation, luteinization
D'Souza et al., 2008	Not done	Not done	dLck-iCre	CD8 T-cell activation, proliferation and survival
Matsushita et al., 2009	Not done	Not done	Col2a1-Cre and Prx1-Cre	Lineage specification of osteo-chondroprogenitor, osteoblast
Srinivasan et al., 2009	Not done	Not done	Tie2-Cre	Endothelial cell proliferation, migration during angiogenesis
Imamura et al., 2010	Not done	Not done	Nestin-Cre	Cortical brain development
Blasco et al., 2011	E1 ≥ E2	Yes	Cre-adenoV + inducible Cre-ERT2	K-Ras induced tumors in lungs, mice death upon KO in adulthood
Sebastian et al., 2011	Not done	Not done	Col2a1-Cre	Growth of cartilaginous skeletal elements, synchondrosis closure
Fyffe-Maricich et al., 2011	Not done	Not done	hGFAP–Cre+ NG2–Cre (= Cspg4Cre)	Differentiation/proliferation of oligodendrocytes, myelination
He et al., 2011	E1 > E2	Yes	Mx1-Cre	Osteoclast differentiation, adhesion, migration, bone resorption
Satoh et al., 2011b	Not done	Not done	Nestin-Cre	Brain development, behavior
Satoh et al., 2011a	E2 > E1	Yes	Nestin-Cre and EIIA-Cre	Social behaviors and learning disabilities
Kehat et al., 2011	E1 ≥ E2	Yes	Nkx2.5-Cre	Cardiac hypertrophy, lengthening vs thickening of myocytes
Otsubo et al., 2012	E2 > E1	Yes	Nestin-Cre	Responses to pain models
Chan et al., 2013	Not done	Not done	Mx1-Cre	Hematopoietic stem cells proliferation, differentiation, aplasia
Staser et al., 2013	Not done	Not done	Mx1-Cre	Hematopoietic stem cells proliferation and differentiation (HSPCs)
Richardson et al., 2015	E1 ≥ E2	Yes	Lyz2-Cre	Proliferation of bone marrow progenitors, macrophages induction
O'Brien et al., 2015	Not in all	Not in all	Nav1.8-Cre	Inflammatory pain, sensory neurons proliferation-differentiation
Chen Z. et al., 2015	Not done	Not done	Osx-Cre	Chondrocyte terminal differentiation, enchondromas

“*ERK1/2 ratio” and “Effect on phospho-ERKs” (2nd and 3rd column) are presented as in Table 1.The promoters driving Cre-recombinase in mice are presented in 4th column. Upon crossing these mice with erk2^floxed∕floxed^ mice, erk2 gene will be invalidated in the tissues expressing the recombinase. Only the main phenotypes studied are presented (last column)*.

#### *erk2* disruption

Since the absence of ERK2 without artificial compensation is lethal for mice, hemizygote disruption (ERK2^+∕−^) and hypomorphic mice were generated. The hypomorphic animals expressed about 20–40% less ERK2 than wild-type ones due to insertion of the neomycin resistance cassette in the 5-prime region of the *erk2* gene (Satoh et al., 2007). The mere presence of the neo cassette reduced ERK2 expression, but unfortunately the active ERK levels were not presented in this study. Nonetheless, gene dosage was indirectly demonstrated since it was impossible to generate an animal expressing only a single hypomorphic allele of ERK2 whereas a smaller increment in ERK2 expression via a single wild-type ERK2 allele was sufficient to sustain life (Satoh et al., 2007). Spatial working memory was normal in animals expressing less ERK2, however mutant mice showed a deficit in long-term memory in classical fear conditioning (Satoh et al., 2007). Lips et al. compared hemizygote disruption of ERK2 with total disruption of ERK1 in ischemic injury studies. Global ERK was immuno-precipitated from ERK2^+∕−^ and ERK1^−∕−^ hearts to measure kinase activity on MBP substrate (Lips et al., 2004). Mice lacking both alleles of ERK1 or only one allele of ERK2 presented very similar levels of ERK activity in hearts as observed in the western blots presented, however these authors claim that only ERK2^+∕−^ mice presented an increased infart area in heart after ischemic injury. The infart surface represented the 30% of the heart in WT mice and it increased up to the 40% ERK2^+∕−^ mice. Considering that mouse hearts express more ERK2 than ERK1 in their study, they may have missed a small difference in ERK immuno-precipitated kinase activity that could explain the lack of statistically significant effect in ERK1^−∕−^ mice compared to the mild effect in ERK2^+∕−^ (Lips et al., 2004). In an independent study from the same laboratory, neither mice lacking both alleles of ERK1 nor mice lacking one allele of ERK2 showed a reduction in pathologic or physiologic stimulus-induced cardiac growth (Purcell et al., 2007). In that study, the impact of gene disruption on phospho-ERK level was negligible and could explain this lack of effect (Figure 1B of Purcell et al., 2007). However, expression of the phosphatase DUSP6 reduced markedly ERK activity in the heart and predisposed mice to heart decompensation and failure (Purcell et al., 2007).

Chen and co-workers have invalided *erk1* and *erk2* genes in mouse embryonic stem cells (ESCs) by Talen or Crisp/Cas9 technologies (Chen H. et al., 2015) (respectively Transcription Activator-Like Effector Nucleases and Clustered Regularly Interspaced Short Palindromic Repeats). These authors established readily *erk1*^−∕−^ and *erk2*^−∕−^ cells. However, double disruption of *erk1* and *erk2* genes was impossible unless ERK1 expression was induced from a cDNA stably integrated. MEK inhibition by small chemical inhibitors is known to promote self-renewal of embryonic stem cells, here ERK expression irrespective of the isoform, and ERK kinase activity were demonstrated to be necessary for ES-cells self-renewal and genomic stability (Chen H. et al., 2015).

#### Tissue specific *erk2* disruption

Tissue specific disruption of *erk2* is necessary to bypass early embryonic lethality. For this purpose exon 3 of ERK2 was surrounded by two loxP sites, and upon expression of the Cre recombinase, exon 3 was deleted to generate a non-functional ERK2 protein (Fischer et al., 2005). Lines of mice expressing Cre recombinase in a tissue at a specific stage of development were crossed with mice harboring ERK2 gene with loxP sites on each side of exon 3 (in Table 2 mice are listed by the promotor driving Cre expression, which defines the cells were recombination occurs). Alternatively, the recombinase can be ubiquitously expressed, but its expression can be pharmacologically switched on. For example tamoxifen has been shown to activate CRE in a timely fashion in Cre-ERT2 mice. Indeed, in adult Cre induction by tamoxifphen led to death by multiple organ failures upon deletion of ERK2 in mice already lacking ERK1 constitutively (Blasco et al., 2011). Finally, Cre recombinase can be expressed by viral infection of airway cells with adenoviruses encoding Cre (Blasco et al., 2011).

Out of 19 publications presenting tissue-specific disruption of *erk2*, we shall limit our detailed analysis to the 4 studies where ERK1 was shown to be more expressed than ERK2 in targeted cells, and the 5 studies where ERK2 was shown to be more expressed than ERK1 (ratio evaluated by western-blot displaying phospho-ERK levels, Table 2). In other publications, the expression ratio between isoforms cannot be evaluated, impeding proper isoform-assignment to the observed phenotypes.

Among cells expressing somewhat more ERK1, Blasco et al. indicate that lung tumor development driven by K-Ras^G12V^ is not impaired upon invalidation of a single isoform; however disruption of both ERK1 and ERK2 appears necessary to block tumor progression (Blasco et al., 2011). Richardson et al. describe that macrophages can proliferate and differentiate by expressing either ERK1 or ERK2. In mice expressing ERK1, removal of *erk2* gene by Cre-recombinase does not change the profile of macrophages, however in mice lacking ERK1, the only macrophages that were encountered were those that did not lose ERK2 (failure of Cre-recombinase to invalidate *erk2* gene; Richardson et al., 2015). In osteoclasts, He et al. (2011) have demonstrated that ERK1 is markedly more expressed than ERK2 and only *erk1*^−∕−^ osteoblast display a clear reduction of the global ERK activity. Indeed only in *erk1*^−∕−^ cells they observed that the ERK substrate p90RSK is less phosphorylated and osteoclast differentiation and bone resorptive activity is reduced. Kehat et al. (2011) have shown that global invalidation of ERK1 and ERK2 was necessary to regulate the balance between eccentric and concentric growth of the heart. They did not assess the contribution isoforms to regulate this balance.

Focusing on ERK2-targeted cells/tissues that express more ERK2, for example, in granulosa cells of mouse ovaries, Fan et al. showed that invalidation of ERK2 or ERK1 partially reduced global ERK activation whereas invalidation of both isoforms abrogated ERK activity (Figure S1A of Fan et al., 2009). Then they demonstrated that maturation of ovocytes and birth of mice pups was abrogated only when both isoforms were invalidated. Interestingly, oocyte maturation occurred nearly normally when global ERK signaling in granulosa cells was provided either by a single ERK1 allele or two alleles of *erk2* (no experiment with only one allele of *erk2* was presented). Considering the care of these authors to show global activation levels in different mice lines, these results clearly argue for a redundant role of ERK isoforms in mouse granulosa cells (Fan et al., 2009).

Satoh et al. invalidated *erk2* in mice brain and observed abnormal social behavior of these mice and impairment of long-term memory (Satoh et al., 2011a). *erk2* disruption led to increased ERK1 activation measured by anti phospho-ERK antibody. To study ERK1 contribution, these authors injected a MEK inhibitor intraperitoneally and observed that 1 h later the levels of phospho-ERKs in the hippocampus, cortex and cerebellum were reduced. In animals lacking ERK2, the inhibitor did not modify the phenotype observed, this result was interpreted by the authors as a failure of ERK1 to drive these behavioral and memory features (especially because ERK1 was over-activated in these brain regions of *erk2*^−∕−(brain)^ mice; Satoh et al., 2011a). An alternative explanation could be that a minimal threshold of global ERK activity for phenotype was already reached in animals lacking ERK2, impeding further behavioral damage. Indeed these authors did not show that the limit of behavioral damage could be increased further than what was observed after *erk2* disruption. With the same model, Ostubo et al. studied the nociceptive response in mice and also implicated ERK2 but not ERK1, again because pharmacological inhibition did not increase the *erk2*^−∕−(brain)^ phenotype (Otsubo et al., 2012). However, in the same laboratory, Satoh et al. showed that the sole deficiency of ERK2 in mice disrupted mildly brain development, however further invalidation of ERK1 aggravated the phenotype leading to death within 1 day after birth (the pups failed to breast-feed in double knock-out mice). In these brain areas, ERK2 was more expressed than ERK1. Hence with this model of single vs. dual invalidation, these authors conclude that total ERK activity, and not a specific ERK isoform, governs cellular behaviors to ensure proper brain development (Satoh et al., 2011b). Hamilton and co-workers invalidated ERK2 expression in mouse embryonic stem cells that express more ERK2 and showed that cells lacking ERK2 displayed enhanced self-renewal capacity and remained even more undifferentiated (Hamilton et al., 2013). Interestingly, these phenotypes were reversed by re-expression of either ERK1 or ERK2 arguing again in favor of isoform redundancy.

In the remaining 13 publications that study consequences of *erk2* gene disruption by recombinase in a specific tissue, the ratio between isoforms was not presented. However, 6 of these studies aimed at determining the role of global ERK activity, not isoform contributions in biological processes (Fischer et al., 2005; Matsushita et al., 2009; Srinivasan et al., 2009; Sebastian et al., 2011; Chan et al., 2013; Chen Z. et al., 2015), and 4 studies demonstrated that there is virtually no phenotype in single mutants whereas in the double *erk1*+*erk2* knockout phenotypes are exacerbated, re-enforcing the notion of isoform functional redundancy (Imamura et al., 2010; Satoh et al., 2011b; Ishii et al., 2012; Staser et al., 2013). Three remaining publications conclude for a specific role of ERK2 (out of 13 that did not quantify isoform ratios), however from our point of view, the lack of measurement of ERK1/ERK2 ratio does not allow to draw clear cut conclusions (D'Souza et al., 2008; Fyffe-Maricich et al., 2011; O'Brien et al., 2015). Furthermore, in a given tissue Cre recombinase expression may not invalidate alleles in all cells. Therefore, it is mandatory to measure to which extent global ERK activity is effectively decreased following mice crossings. This is particularly obvious in light of the minimal decrease of ERK activity observed by O'Brien et al. in dorsal root ganglia of mouse invalidated for ERK2 (Figure 5A of O'Brien et al., 2015). In that study, only phenotypes of animals lacking both isoforms allow to conclude that ERK is essential for sensory neuron biology.

#### Conclusions from *erk1, erk2* gene disruptions in mice

When taking into account the isoform ratio and effectiveness to reduce global ERK activity, the studies presenting disruption of *erk1/2* alleles in mice, give overwhelmingly credit toward functional redundancy between ERK1 and ERK2.

### Isoform loss in vertebrates

As described above, silencing experiments in cells and gene disruption in mice provide strong arguments for a functionally redundant role of ERK isoforms, furthermore, deletion of *erk1* gene in laboratory mice is compensated by increase of endogenous ERK2 activity to allow normal development and reproduction. Therefore, one can wonder why all mammals analyzed so far have kept expression of both ERK1 and ERK2? We have used another approach to investigate this question by obtaining insights from vertebrate evolution (Busca et al., 2015).

First it is striking that cartilaginous fishes, birds and frogs do not possess *erk1* genes, confirming that vertebrate life is compatible with total loss of one isoform. All other vertebrates analyzed so far possess *erk1* and *erk2* genes. The high expression level of ERK proteins, and the availability of anti-phospho ERK antibodies allowed to determine expression of isoforms in all vertebrates (the phospho ERK epitope is 100% conserved in vertebrates; Busca et al., 2015). In reptiles, two species of turtles express both ERK1 and ERK2, but crocodiles do not express ERK1 at detectable levels, and more surprisingly no ERK2 protein was detected in squamates (snakes lizards and geckos). In lizards, ERK2 was not present in all brain areas studied and all adult tissues tested (it is important to note that ERK is highly expressed in the brain). To confirm unambiguously this observation, only siRNA targeting ERK1 reduced ERK protein expression in lizard embryo fibroblasts. As controls, the pools of siRNA targeting ERK1 or ERK2 were able to reduce the level of their respective RNAs. The strength of *erk* proximal promoters were compared (about 1 kb upstream initiating ATG). In lizards, *erk2* promoter is markedly weaker than *erk1* promoter, whereas it is the opposite for mouse promoters (Busca et al., 2015). Therefore, in vertebrates, ERK signal can be provided either by both isoforms or only by ERK1 or ERK2. One reason explaining the predominance of isoforms lies at least on the strength of their respective promoters.

The question can be turned up-side down: why most vertebrates express functionally redundant ERK isoforms? As presented above, ERK1 and ERK2 isoforms appeared about 400 million years ago in the course of whole genome duplication (WGD) in early vertebrates. Most duplicate genes are lost progressively during evolution of species; however after a WGD the loss of duplicated genes is slower than gene loss after local duplication (Blomme et al., 2006). Indeed, from studying WGD in paramecium, it was shown that duplicates with no divergent functions can be kept for of millions of years; interestingly genes were shown to be kept longer as duplicates if they are highly expressed and/or if they belong to signaling cascades (Aury et al., 2006; Brunet et al., 2006). Therefore, considering that ERK1 and ERK2 are both highly expressed and signaling molecules, they are good candidates to be kept as duplicates for many millions of years, even without being endowed with novel functions nor sub-functionalization. Nonetheless, duplicates can still be lost when dosage is provided by one isoform, already cartilaginous fishes, birds and frogs have lost the gene one *erk* isoform. Will a wild mammal lacking one *erk* gene ever be found?

Phylogenic studies revealed that ERK1 and ERK2 nucleotide sequences evolved at similar rates, but the sequences of ERK1 proteins evolved faster than those of ERK2 proteins. This could indicate a less important/dispensable role of ERK1. However, it should be noted that among all vertebrate MAP kinases, ERK2 sequences are the most stable ones, and the rate of ERK1 sequences evolution is still very low, in the range measured for all p38-MAP kinases (Li et al., 2011). By aligning sequences on ERK1's 3D structure, it was shown that the few positions displaying variable amino-acids among mammalian ERK1s are all located on the back of the kinase, away from domains that bind to activators and substrates and away from kinase effector domains. Therefore, positions essential for all known functions are invariant in vertebrate ERK1 and ERK2 sequences, arguing against isoform-specific functional differences. In most vertebrates, and especially in mammals, *erk2* genes are much larger than *erk1* (15 fold) providing a structural explanation (and not a functional one), for the slower evolution rate of ERK2 protein sequences, since larger genes can recombine more easily to purify mutations during evolution (Marais et al., 2005; Liao et al., 2006).

Overall these phylogenic studies accredit the hypothesis that ERK1 and ERK2 are functionally redundant kinases, whose protein domains essential for function remain extremely conserved across evolution.

### Replacement of *erk2* in mice

In the three studies that have described the lethality of *erk2* gene disruption in mice (Hatano et al., 2003; Saba-El-Leil et al., 2003; Yao et al., 2003), it was suggested that ERK1 is insufficiently expressed at some developmental stages to complement ERK2 loss to convey properly ERK signaling. To validate this hypothesis, Frémin and co-workers intended to express *erk1* cDNA in the first exon of endogenous mouse *erk2* gene, which should have allowed expression of ERK1 at the levels of endogenous ERK2 since the half-life of mouse ERK mRNAs and mouse ERK proteins were shown to be very similar (Schwanhausser et al., 2011). Unfortunately, neither *erk1* cDNA, nor a control *erk2* cDNA, drove expression to levels near those of endogenous *erk2*, precluding the use of these constructs to generate mice.

However, these authors generated transgenic mice expressing ERK1 under the control of the ubiquitously expressed chicken beta-actin promoter (Frémin et al., 2015). In these mice, transgenic-ERK1 was expressed in all early embryonic stages to all adult tissues at elevated levels. Upon crossing these mice with *erk2*^+∕−^ mice, it was possible to obtain mice lacking both alleles of *erk2* at Mendelian rates. They found that mice lacking ERK2 lived and reproduced normally, establishing that ERK1 protein can replace ERK2 throughout all mammalian life. It is very interesting to note that ERK1 seemed to be more expressed in transgenic-ERK1 animals than ERK2 in wild-type animals, however the global level of ERK activity was very similar in both animal lines (Frémin et al., 2015). In few tissues such as the heart, transgenic-ERK1 expression was much higher than ERK2 expression in WT-animals, but global active ERK seemed nearly identical in transgenic-ERK1 animals and WT ones (Figure S3H in Frémin et al., 2015). This observation confirms the high resiliency of ERK signaling to perturbations such as over-expression of one component, at least in the absence of challenging stimuli. In mouse embryo expressing transgenic-*erk1* in *erk2*^f^^∕f^ background, the highly expressed ERK1 protein did not change the expression of endogenous ERK2 but it captured most of the activation from upstream MEK, illustrating clearly again the resiliency of the pathway (ERK2 activation is markedly diminished, Figure S3C in Frémin et al., 2015). This observation demonstrates once more the equivalence of ERK1 and ERK2 isoforms to receive MEK activation. Furthermore, upon crossing animals missing different alleles of ERK isoforms, these authors have demonstrated that the extent of placental and embryonic development is strictly correlated to global ERK activity (Frémin et al., 2015).

The lethality of *erk2* loss in mice can be compensated when ERK1 expression is increased in all tissues. Then, there is a tight correlation between global ERK activity and the phenotypes observed, irrespectively of the isoform expressed. Taken together these observations demonstrate functionally redundant roles for ERK1 and ERK2 by this gene-replacement strategy.

## Conclusions/perspectives

Deregulation of the ERK signaling cascade is devastating for many patients. Many clinical trials with inhibitors of Raf, MEK and ERK are ongoing to overcome cancer, stressing the importance in understanding all aspects of this signaling cascade.

At the level of Ras, several cancers are triggered almost exclusively via mutation of a single isoform such as only K-Ras mutations found in pancreatic cancers for example (Bryant et al., 2014). At the level of Raf, pharmacological inhibitors targeting a single isoform have been approved to treat patients. At the level of MEK, distinct retro-phosphorylations provide a rationale for having two isoforms. Overall, isoform specificities play a significant role in this pathway, therefore the quest in understanding why two isoforms convey ERK signaling appears of great importance.

With regards to protein sequences, ERK1 and ERK2 are very stable across evolution, with variations in amino-acids only at positions that are neutral for function. Presently, neither ERK isoform-specific agonists, nor isoform-specific substrates have been found. These observations point again toward functional redundancy of ERK1 and ERK2. However, these kinases are not redundant *per se* since disruption of *erk2* without compensation by ERK1^transgenic^ is lethal in mice. Indeed, ERK1 and ERK2 are expressed ubiquitously but not at the same level in most tissues. Clear differences between ERK1 and ERK2 are obvious at the gene level. For example, in mammals *erk2* genes are on average 15 times larger than *erk1* genes, and in mice the 3-prime UTR of *erk2* is both 6 times larger than *erk1* and possess an alternative poly-adenylation site. These differences indicate that ERK2 has the capacity to be more exquisitely regulated than ERK1. Apart from these differences in expression levels, we cannot exclude that very fine specializations exist between ERK1 and ERK2, such as a slightly stronger affinity for a substrate or an interactor protein. Only replacement of all exons of one isoform with those of the other isoform may uncover these fine differences; however it might be very complicated to draw conclusions if the replaced exons modify protein stability and change even slightly the global ERK quantity/activity ratio!

The studies presented here using knock-down approaches or gene disruptions overwhelmingly demonstrate a direct correlation between a biological consequence and the global level of ERK activation, irrespectively of the isoform. In cells with complex fate such as a balance between proliferation and differentiation, not all combinations have been studied so far. For example one would have liked to compare knock-out of a single *erk2* allele with knock-out of both *erk1* alleles; pending these two conditions would decrease global ERK activation to the same extent. Among three add-back studies presented here, one concluded that transfection of either ERK1 or ERK2 could reverse the phenotype of ERK2 loss, whereas the two other studies were inconclusive since it was not demonstrated that individual ERK transfections re-established the active ERK levels. In our hands it has proven difficult to re-express ERK that can be activated as efficiently as endogenous ERK (efficiency measured as the percentage of the transfected kinase that is effectively activated). Similarly, Meloche and co-workers did not succeed to express ERK1 or ERK2 at normal levels from cDNA inserted in mouse *erk2* locus (Frémin et al., 2015). Further work is needed to understand the means by which ERK proteins are highly expressed and regulated.

In more than 75 studies, the decrease of ERK expression was shown to trigger biological phenotypes, therefore it is surprising that over-expression of ERK1 in mice does not cause apparent phenotypical changes (Frémin et al., 2015)! We predict that under challenging conditions, phenotypes will be discovered in this ERK1-transgenic model. In fact, despite the elevated quantity of ERK1 in these mice, global ERK activation level seemed normal due to the robustness of the pathway via multiple retro-inhibitions. However, ERK quantity needs to be regulated, at least under challenging conditions. For example *erk2* gene amplification in humans was demonstrated to be the cause of tumor resistance to cancer treatment (Ercan et al., 2012). Therefore, more research is needed to understand the relevance of global ERK quantity for proper signaling under challenging conditions.

Lack of ERK is lethal in vertebrates but mice have been generated without ERK1 protein or without ERK2 protein, and tetrapods (vertebrates with four limbs) express either both ERKs or only ERK1 or only ERK2. In many studies, a direct correlation between the global quantity of ERK activation and phenotypical consequences has been established. Taken together, these data strongly suggest that ERK1 and ERK2 are functionally redundant.

## Author contributions

PL, RB work in the laboratory of JP, in the IRCAN institute of Nice. PL wrote the main body of the text that was amended by RB and corrected in detail by JP.

### Conflict of interest statement

The authors declare that the research was conducted in the absence of any commercial or financial relationships that could be construed as a potential conflict of interest.
